# Comparative Analysis of Cell Metabolic Activity Sensing by *Escherichia coli* *rrnB* P1-*lux* and Cd Responsive-*Lux* Biosensors: Time-Resolved Experiments and Mechanistic Modelling

**DOI:** 10.3390/bios12090763

**Published:** 2022-09-16

**Authors:** Eva Delatour, Christophe Pagnout, Marie L. Zaffino, Jérôme F. L. Duval

**Affiliations:** 1 Université de Lorraine, CNRS, LIEC (Laboratoire Interdisciplinaire des Environnements Continentaux), UMR7360, Campus Bridoux, F-57070 Metz, France; 2 Université de Lorraine, CNRS, LIEC, UMR7360, F-54501 Vandoeuvre-lès-Nancy, France

**Keywords:** bioluminescence, whole-cell biosensors, metals, bioavailability, medium nutritional quality, signal dependence on time

## Abstract

Whole-cell bacterial sensors are used in medical/environmental applications to detect chemicals, and to assess medium toxicity or stress. Non-specific constitutive biosensors generally serve the latter purpose, whereas chemical detection is performed with biosensors involving a specific chemical-inducible promoter. Herein, we show that functioning principles of specific and non-specific whole-cell biosensors are not exclusive as both can probe modulations of cell metabolic activity under stressing conditions. The demonstration is based on (i) *time-resolved* measurements of bioluminescence produced by constitutive *rrnB* P1-*luxCDABE Escherichia coli* biosensor in media differing with respect to carbon source, (ii) theoretical reconstruction of the measured signals using a here-reported theory for bioluminescence generated by constitutive cells, (iii) comparison between time-dependent cell photoactivity (reflecting metabolic activity) retrieved by theory with that we reported recently for cadmium-inducible P*zntA*-*luxCDABE E. coli* in media of similar compositions. Whereas signals of constitutive and non-constitutive biosensors differ in terms of shape, amplitude and peak number depending on nutritional medium conditions, analysis highlights the features shared by their respective cell photoactivity patterns mediated by the interplay between stringent response and catabolite repressions. The work advocates for the benefits of a *theoretical interpretation for the time-dependent response of biosensors* to unravel metabolic and physicochemical contributions to the bioluminescence signal.

## 1. Introduction

Whole-cell bacterial sensors are now well-established tools among the arsenal of instruments used by the scientific community for detecting and quantifying chemicals (e.g., drugs [1,2], toxins [3], quorum sensing signaling molecules [4], siderophores [5]), pollutants (e.g., metals [6], organics [7]) or microbial pathogens [8] in biological media [9] or environmental samples [10,11], and for addressing the toxicity of contaminants [12]. They offer an elegant alternative to conventional physicochemical methods (e.g., extraction, chromatography) due to their cost, ease of use, specificity and sensivity [13]. Bacterial sensors generate a measurable signal (e.g., luminescence, fluorescence) following the expression of a reporter gene (e.g., *lux* and *gfp*, respectively) either naturally present in the bacterial strain or introduced by genetic engineering downstream of a promoter [13]. Depending on the manner in which this promoter is activated, whole-cell bacterial sensors can be classified into three distinct categories [12]: non-specific (or general) toxicity biosensors, such as the historical and commercially available Microtox^®^ [14,15], semi-specific biosensors responding to a given stress [12,13,16,17,18], and specific biosensors whose output signal is triggered by a given chemical compound that actuates a tightly regulated promoter fused upstream of the reporter gene [6,13,18,19].

Taking the example of luminescence (*lux*)-based whole-cell biosensors of interest in this work, non-specific toxicity biosensors involve a promoter that is constitutively expressed under normal conditions and fused to a *lux* operon [20]. These biosensors report a decrease in bioluminescence as a result of a reduction in or suppression of the promoter’s transcription following a decrease in cell metabolic activity induced by harmful effects caused by a toxicant or a mixture of toxicants [21]. Illustrative examples of such promoters are *rrnB* P1 that controls the expression of one of the seven rRNA operons in *E. coli* [22,23] or the promoters P*cpc* or P*psbA* that control the expression of the main components of the photosynthetic apparatus in cyanobacteria [24]. Semi-specific luminescent biosensors produce a signal in response to harmful chemicals and/or to stressing medium conditions upon activation of defence mechanism(s) against the produced stress. Here, the biosensor signal is induced by the expression of a *lux* operon triggered by a promoter that is activated by the stress, e.g., damage of DNA, proteins or membrane, or production of reactive oxygen species (ROS) [12,17,25,26]. Lastly, the functioning of specific whole-cell *lux*-based biosensors relies on the activity of a promoter that is stimulated by a given target chemical whose intracellular sensing is ensured by a regulatory protein and by the subsequent promoter expression. Historical examples of specific promoter-*lux* assemblies include *tbuA1p-luxAB, arsRp-luxAB, merTp*-*luxCDABE,* *zntAp-luxCDABE* or *alkAp*-*luxCDABE* for the detection of benzene, arsenite, mercury, cadmium, and DNA-alkylating agents, respectively, to quote only a few [13].

The working principles of non-specific, semi-specific (stress-responsive) and specific (chemical-responsive) whole-cell biosensors are not exclusive in practice. Indeed, the possible stress induced by a chemical to be detected by a biosensor specifically designed for that purpose, may lead to a decrease in or even suppression of the cell signal. In turn, this reduces detection sensitivity and diminishes the capability of the biosensor to probe quantitatively the occurrence of chemicals in a solution. Similar effects may be produced without invoking chemical-induced stress, e.g., deficiencies of nutrients in the extracellular medium, a lack of cofactors required to sustain cell activity and metabolism, a poor quality of the supplied food, or even interactions of nutrients with targeted chemical with a resulting decrease in food and/or chemical bioavailability. These undesired but sometimes unavoidable processes can severely inhibit the expression of the reporter gene and impact on the sensing function of the biosensor. Methodologies to circumvent these undesired cross-reactivities affecting specificity and/or sensitivity of whole-cell biosensors, exist. They consist (i) in the use of bioreporters combining functional properties of non-specific, semi-specific and/or specific biosensors with the possible accommodation of several reporter genes in a single sensing cell [12,27], (ii) in the reading of a signal output delivered separately by whole-cell biosensors that differ with respect to their specificity level [23], (iii) in the recourse to biosensors designed for nutrient analysis [28], or (iv) in the construction of microfluidic device for parallel single-analyte or simultaneous multi-analyte detections by coupling multiplex biosensors [7,10].

This ensemble of experimental designs actually palliates the difficulty to address theoretically the multiple extracellular and intracellular factors that control the time-dependent response of whole-cell biosensors and their overall performance. For the sake of illustration, quantifying the concentration of (bioavailable) metals from the signal of a metal-responsive (non-constitutive) luminescent biosensor still remains today a formidable challenge. This is explained by the complexity of the intertwined processes that define, e.g., the partitioning of the relevant metal forms at the biosensor/solution interface [29,30,31,32,33,34,35,36,37,38] and the ensuing time-dependent bioluminescence [39,40]. In turn, the analysis remains currently limited to correlative observations that often involve the recourse to calibration media whose nutritional quality is tacitly supposed equivalent to that of the sample of interest. Common strategy further relies on empirical connections between bioluminescence cell output and bulk metal concentrations estimated from equilibrium Biotic Ligand Model (BLM) and, e.g., Visual MINTEQ metal speciation code [13,29,41,42,43,44,45,46]. As extensively commented elsewhere [29,30,31,32,33,34,35,36,37,38,39,40], the BLM-based approach suffers from a number of approximations, e.g., it ignores a priori the possible contribution of labile metal complexes to the flux of bioaccumulated metals [30,32,36,37,38], it misses any rationale of the energetic cell demands required to convert internalized metals into cell signal output [23,39,40], it fails to reproduce metal bioaccumulation kinetics captured by Best’s formalism and extensions thereof [30,31,33,34], and it discards the implications of bulk metal depletion [31,33,34,47] or passive metal adsorption [48] on metal bioavailability and biosensor signal. Similarly, theoretical evaluation of medium toxicity from analysis of non-specific biosensors signal is tied to a successful formulation of cell sensitivity to stress and of the resulting cell ability to maintain (or not) light production under unfavourable conditions. As a result, the analysis of the response of constitutive and non-constitutive biosensors is generally restricted to mere consideration of the only maximal cell signal amplitude [41,42,43,44,45,46] without comprehensive evaluation of the information-rich dependence of the signal on time [40]. The feasibility of current biosensing practice and reliability of corresponding results thus basically rests on the availability of proper controls as any decrease in the overall metabolic activity of a given stress-responsive or chemical-responsive biosensor may produce similar effects to those in the absence of the targeted stress or chemical, which possibly leads to false negative or false positive [12]. Outcomes are therefore often semi-qualitative at best. Clearly, further mechanistic and quantitative information on the biotic and abiotic factors governing bioluminescence emission by whole-cell biosensors is still critically needed.

In their attempt to rationalize and increase predictability of whole-cell biosensors signal, Duval et al. derived a formalism for the time-dependent bioluminescence response of metal-detecting *lux*-based *Escherichia coli* biosensors under conditions where cell concentration remains constant over time and metal depletion from bulk solution is not operational [39]. Theoretical results were successfully confronted against measurements of bioluminescence produced by *E. coli* sensors engineered with pZnt-*lux* plasmid harbouring a Cd-inducible *zntA*-*luxCDABE* gene fusion [23,39,40]. The formalism explains how the dependence of bioluminescence on time is governed by rapidly established steady-state metal biouptake flux, by the metal-mediated kinetics of *lux*-reporter expression, by the ensuing rates of luciferase production/degradation, and the kinetics of photon emission. A remarkable result is that, contrary to modelling reports in the literature [49], bioluminescence is *not* proportional to the concentration of photoactive cells in solution or, equivalently, it is not related linearly to cell photoactivity. Changes in the latter with time reflect the way cells (re)allocate bioavailable energetic resources to sustain light production, cope with or adapt to toxicity/hormesis effects mediated by contaminants or to limitations in essential nutrients [23,39,40,48]. In line with this theory, the cell signal may then take the form of multiple, well-defined successive peaks whose defining patterns (amplitude, number, shape, time-positioning) are not only dependent on metal concentration but also on the kinetic settings of the metabolic pathways over the course of time [23,40].

As a follow-up of these results, we provide here a comprehensive and parsimonious theory for modelling the full time-dependence of bioluminescence signal generated by non-specific constitutive *rrnB* P1-*luxCDABE Escherichia coli* biosensor. The structure of the obtained theoretical expression is validated by the confrontation to time-resolved bioluminescence measurements in aqueous media with variable contents of carbon in the form of glucose and/or xylose. Fundaments of the theory are further supported by comparison with results we recently reported for Cd-inducible *luxCDABE-*based *E. coli* under similar cell incubation conditions [40]. Despite the differentiated shapes of the constitutive and non-constitutive cell responses with time, the analysis evidences the common features shared by their time-dependent cell photoactivities estimated from proper deconvolution of the signals. Remarkably, we show how the responses of both biosensors, despite their different modes of promoter activation, reflect the same processes that govern the metabolism of amino acids and carbon required to sustain light production. Differences between constitutive and non-constitutive cell signal patterns originate from the here-formulated impacts of whole-cell biosensor conditioning prior to bioluminescence measurement, and from the extent of the long-term signal extinction caused by the inhibition of luciferase production and activity.

## 2. Materials and Methods

### 2.1. Luminescent Whole-Cell Bacterial Reporters

The non-specific whole-cell bacterial sensor adopted in this work was constructed from *Escherichia coli* BW25113 strain obtained from the Coli Genetic Stock Center at Yale University [50]. Construction was realized by transforming BW25113 strain with the plasmid pET28 *rrnB* P1*-luxCDABE* [51] where the expression of *luxCDABE* operon is controlled by the ribosomal RNA *rrnB* P1 promoter. This plasmid was kindly provided by Kulbachinskiy A. and Esyunina D. from the Institute of Molecular Genetics, Moscow. Time-resolved bioluminescence data collected on *rrnB* P1*-luxCDABE* BW25113 sensor were systematically compared with those we reported elsewhere [40] for the Cd-responsive p*ZntA*-*luxCDABE* BW25113 whole-cell biosensor under similar incubation conditions (specified in §2.2). The latter specific Cd-responsive biosensor was constructed with the plasmid pUCD615 p*Znt-luxCDABE*. To ensure the relevance of the comparison, bioluminescence responses of the non-specific constitutive *rrnB* P1-*luxCDABE* biosensor were measured in media containing Cd ions in the concentration range 0–20 nM which excludes Cd-mediated toxicity effects [23,40,48].

### 2.2. Cell Growth Condition, and Nutritive Medium Composition in Bioluminescence Assays

The procedure adopted here to prepare the suspensions of biosensors is similar to that detailed in [40] except for a few modifications. In detail, the cryo-preserved (−80 °C) constitutive bacterial cells were inoculated on LB-agar agar supplemented with 30 µg/mL of kanamycin and incubated at 37 °C for 24 h. Isolated colony was transferred to a 125 mL erlenmeyer flask containing 20 mL of LB medium (10 g/L casein peptone, 5 g/L yeast extract and 10 g/L NaCl, Fisher Scientific™) supplemented with 60 µL kanamycin 10 mg/mL. Samples were then incubated at 37 °C for 9 h under agitation at 160 rpm. Pre-cultured cells were used to inoculate (at 1:100 dilution) 100 mL of LB medium with 300 μL of kanamycin 10 mg/mL, after which cells were incubated overnight at 37 °C under agitation at 160 rpm. The OD_600nm_ of the thus-prepared bacterial suspensions was measured, and samples were then subdivided into 16 aliquots of 5 mL. These 16 aliquots were washed twice by centrifugation (7000× *g*, 3 min) with nGGM medium (MOPS 40 mM (Acros organics), MgCl_2_ 1 mM (Sigma-Aldrich), NH_4_NO_3_ 12.5 mM (Merck), 10 mM KNO_3_ (Normapur^®^), 5 mM K_2_SO_4_ (Normapur^®^), 0.068 mM CaCl_2_ (Prolabo), 5 mM β-glycerophosphate (Sigma), pH 6.8 adjusted by addition of 0.1 M NaOH) supplemented with tryptone (1% *v*/*v*) as amino acids source (Euromedex), and glucose and xylose (Sigma-Aldrich) at different concentration ratios (100/0, 50/50, 30/70, 20/80, 18/82, 16/84, 14/86, 12/88, 10/90, 6/94, 4/96, 2/98). In the following section, we denote *x* as the ratio between glucose concentration and sum of glucose and xylose concentrations, the explored conditions thus corresponding to *x* = 1, 0.5, 0.3, 0.2, 0.18, 0.16, 0.14, 0.12, 0.1, 0.06, 0.04, and 0.02. Cell suspensions were resuspended in these media to obtain a final optical density of 2.0 at 600 nm. The 100% glucose and 100% xylose reference conditions correspond to 0.5% (*m*/*v*) glucose and 0.5% (*m*/*v*) xylose concentrations in the medium, respectively.

### 2.3. Bioluminescence Measurements

Similarly to [40], bioluminescence measurements were performed in a 96-well microplate (NuncTM, Thermo ScientificTM, Illkirch, France) with a SAFAS Xenius luminometer (SAFAS, Monaco). Wells were filled with 70 µL of milli-Q ultrapure water, 10 µL of nGGM medium, 10 µL of bacterial culture prepared according to §2.2, and 10 µL of Cd(NO_3_)_2_ (Fluka) to obtain a final total concentration range of Cd(II) from 0 to 20 nM. This range corresponds to a linear response of the non-constitutive Cd-responsive cells with total Cd concentration, and to the absence of metal-induced toxicity effects [40,48]. In turn, tryptone concentration adopted for the bioluminescence assays was 0.1% (*v*/*v*), and that of the nGGM-glucose/xylose medium corresponded to a 5-fold dilution of the nGGM glucose/xylose solution prepared as detailed in §2.2. Luminescence was measured at 490 nm every 5 min for 48 h at 25 °C, with each measurement preceded by orbital shaking for 10 sec (3 mm amplitude at 10 Hz frequency). We further verified that the optical density at 600 nm remains constant at a value of 0.2 over the entire duration of the luminescence assays, thereby ensuring that the *total* cell concentration is independent of time. Below, we identify the mathematical similarities between here-elaborated time-dependent bioluminescence expression for constitutive biosensors and the one reported in [39,40] for their metal-responsive analogues.

## 3. Theory

### 3.1. Kinetics of Bioluminescence Emission by Non-Specific Lux-Biosensors Involving a Constitutive Promoter

In the following developments, we consider an aqueous medium of volume $V_{T}$ containing *lux*-based bacterial sensors producing bioluminescence constitutively. We denote as $N_{c}\left(t\right)$ the number of photoactive cells at time *t*, and the corresponding concentration of photoactive bacteria is defined by $c_{p,c}\left(t\right)=N_{c}\left(t\right)/V_{T}$ (m^−3^), where the subscript c stands for ‘constitutive’. The overall concentration of luciferase produced in the entire volume $V_{T}$ at *t* is given by $S_{a}\phi_{\mathrm{Lu},c}\left(t\right)c_{p,c}\left(t\right)$ (in mol m^−3^) where $\phi_{\mathrm{Lu},c}\left(t\right)$ (in mol m^−2^) is the intracellular concentration of luciferase at *t* expressed per unit cell surface, and $S_{a}$ is the cell surface area (m^2^). Accordingly, the rate of luciferase production per unit solution volume is written $S_{a}d\left[\phi_{\mathrm{Lu},c}\left(t\right)c_{p,c}\left(t\right)\right]/dt$ (in mol m^−3^ s^−1^) where $S_{a}$ is set independent of time. At any *t*, this quantity must satisfy the mass balance condition which we write in the simple form

(1) $$S_{a}d\left[\phi_{\mathrm{Lu},c}\left(t\right)c_{p,c}\left(t\right)\right]/dt=k_{f,c}c_{p,c}\left(t\right)/K_{\mathrm{Hi},c}-k_{r,c}S_{a}\phi_{\mathrm{Lu},c}\left(t\right)c_{p,c}\left(t\right)+k_{\mathrm{fo},c}$$

Following Hill’s framework for the practical formulation of protein production rates [39,52,53,54], $K_{\mathrm{Hi},c}$ (in m^−3^) in Equation (1) serves as an indicator for the number concentration of regulating RNA polymerase (per cell volume) required to initiate gene transcription, and $k_{f,c}$ (in mol m^−3^ s^−1^) stands for the kinetic constant of luciferase production (or formation, subscript ‘f’) whose rate at *t* is set proportional to the corresponding concentration $c_{p,c}\left(t\right)$ of radiating cells present in solution [55]. In turn, the quantity $k_{f,c}/K_{\mathrm{Hi},c}$ can be viewed as a measure of the strength of the promoter. The term $k_{r,c}S_{a}\phi_{\mathrm{Lu},c}\left(t\right)c_{p,c}\left(t\right)$ in Equation (1) corresponds to the effective rate of luciferase ‘removal’, which encompasses all processes leading to a reduction in the flux of emitted photons, e.g., luciferase activity inhibition [39], with an associated kinetic constant denoted as $k_{r,c}$ (in s^−1^). Finally, $k_{\mathrm{fo},c}$ is the kinetic constant (in mol m^−3^ s^−1^) featuring any possible basal production of luciferase, independent of cell concentration in solution [52]. As demonstrated in [39], the bioluminescence produced at *t*, denoted as $Lum_{c}\left(t\right)$ (in counts s^−1^), is further given by the following integral equation

(2) $$Lum_{c}\left(t\right)=k_{\nu }S_{a}V_{T}\int_{0}^{t}q\left(t,\tau \right)\frac{d\left[\phi_{\mathrm{Lu},c}\left(\tau \right)c_{p,c}\left(\tau \right)\right]}{d\tau }d\tau$$

where $k_{\nu }$ (counts mol^−1^ s^−1^) is the kinetic constant for photons production per mole of luciferase and $q\left(t,\tau \right)$ is a dimensionless function that quantifies how much bioluminescence at time *t* has decreased for bacterial cells activated at an earlier time *τ*(<*t*) [55], this idea being in line with the well-documented decay kinetics of photons emission by bacterial luciferase [56]. The (dimensionless) factor $q\left(t,\tau \right)$ can be interpreted in different ways, all leading to the mathematical formulation of bioluminescence according to Equation (2) [55]. It reflects either a decrease in the number of photoactive cells in solution (i.e., a fraction of cells is inactive (no emission), whereas another produces light at constant emission rate), a decrease in the emission rate of all individual cells (all producing light with a given decay kinetics), or a combination of these two effects. The key idea remains the mandatory discrimination between time-dependent concentration of photoactive cells and total cell concentration [39,55], the latter being constant over time under the experimental conditions of interest in this work (cf. §2). Retaining the first aforementioned definition for $c_{p,c}\left(t\right)$, the fraction of photoactive cells at time *t* is necessarily connected to the concentration of produced luciferase as governed by the differential Equation (1). For the sake of simplicity, we consider that luciferase generated at time τ emit photons at constant rate $k_{\nu }$ for a duration of $\tau_{q}$ (s) referred to as ‘quenching time’ in [55]. This representation comes to assimilate the function $q\left(t,\tau \right)$ to a gate function with unit value for $\tau \leq t\leq \tau +\tau_{q}$ and with 0 value outside this time interval [39]. Equation (2) then becomes [39]

(3) $$Lum_{c}\left(t\right)=k_{\nu }S_{a}V_{T}\left\{\phi_{\mathrm{Lu},c}\left(t\right)c_{p,c}\left(t\right)-H\left(t,\tau_{q}\right)\phi_{\mathrm{Lu},c}\left(t-\tau_{q}\right)c_{p,c}\left(t-\tau_{q}\right)\right\}$$

where $H\left(t,\tau_{q}\right)$ is the Heaviside function defined by $H\left(t,\tau_{q}\right)=0$ for $t\leq \tau_{q}$ and $H\left(t,\tau_{q}\right)=1$ for $t>\tau_{q}$. Realizing that the emission decay kinetics for luciferase molecule is much faster than the measurement timescale, i.e., $t>>\tau_{q}$ (with t∈ [0–48 h] in this work, and $\tau_{q}$~1 min at most [55,56]), Equation (3) simplifies into

(4) $$Lum_{c}\left(t\right)=k_{\nu }\tau_{q}S_{a}V_{T}d\left[\phi_{\mathrm{Lu},c}\left(t\right)c_{p,c}\left(t\right)\right]/dt$$

Solving Equation (1) and substituting the solution into Equation (4), we obtain after some algebra

(5) $$Lum_{c}\left(t\right)=k_{\nu }V_{T}K_{\mathrm{Hi},c}^{-1}k_{f,c}\tau_{q}c_{p,c}^{\max}\left[{\bar{c}}_{p,c}\left(t\right)+\beta e^{-k_{r,c}t}-k_{r,c}\chi_{c}\left(t\right)⊗{\bar{c}}_{p,c}\left(t\right)\right]$$

where we have introduced the dimensionless concentration of photoactive cells at *t*, ${\bar{c}}_{p,c}\left(t\right)$, hereafter termed cell photoactivity and defined by ${\bar{c}}_{p,c}\left(t\right)=c_{p,c}\left(t\right)/c_{p,c}^{\max}$, with $c_{p,c}^{\max}$ the maximum number concentration (in m^−3^) of light-producing cells the medium can sustain over the whole time-window of the bioluminescence measurements. Stated differently, $c_{p,c}^{\max}$ refers to the medium carrying capacity which obviously depends on the quality and amount of nutriments in the solution. To facilitate comparison with the results reported in [40] for metal Cd-responsive whole-cell biosensors, we adopt the convention ${\bar{c}}_{p,c}\left(t=0\right)=0$. The symbol ⊗ in Equation (5) defines the convolution product operator in the time domain, i.e., $x\left(t\right)⊗y\left(t\right)=\int_{0}^{t}x\left(t-\xi \right)y\left(\xi \right)d\xi$ where $x\left(t\right)$ and $y\left(t\right)$ are dummy functions of time. The time-dependent function $\chi_{c}$ in Equation (5) is given by $\chi_{c}\left(t\right)=e^{-k_{r,c}t}$, and the dimensionless constant β in Equation (5) is further provided by

(6) $$\beta =k_{\mathrm{fo},c}K_{\mathrm{Hi},c}/\left(k_{f,c}c_{p,c}^{\max}\right)$$

We easily infer from Equations (5) and (6) that bioluminescence at t=0 relates to $k_{\mathrm{fo},c}$ via

(7) $$Lum_{c}\left(t=0\right)=k_{\nu }V_{T}K_{\mathrm{Hi},c}^{-1}k_{f,c}\tau_{q}c_{p,c}^{\max}\beta =k_{\nu }V_{T}\tau_{q}k_{\mathrm{fo},c}$$

Equations (5) and (6) are important results of this paper as they explicitly detail how bioluminescence of constitutive whole-cell biosensors evolves with time depending on cell photoactivity profile (${\bar{c}}_{p,c}\left(t\right)$), promoter strength and rate of luciferase production ($k_{f,c}$,$k_{\mathrm{fo},c}$,$K_{\mathrm{Hi},c}$), luciferase inhibition kinetics ($k_{r,c}$), characteristic timescale of luciferase emission ($\tau_{q}$), and medium carrying capacity ($c_{p,c}^{\max}$).

On the basis of Equation (5), explicit analytical expressions of $Lum_{c}\left(t\right)$ can be derived in the scenario corresponding to $\tilde{t}<t<<\tilde{t}+1/k_{r,c}$ where $\tilde{t}$ is the timepoint beyond which medium carrying capacity is reached, i.e., ${\bar{c}}_{p,c}\left(t>\tilde{t}\right)∼1$. Under such conditions, we show that $Lum_{c}\left(t\right)$ decreases linearly with *t* according to

(8) $$Lum_{c}\left(t\right)\approx k_{\nu }V_{T}K_{\mathrm{Hi},c}^{-1}k_{f}\tau_{q}c_{p,c}^{\max}\gamma \left[1-k_{r,c}\left(t-\tilde{t}\right)\right]$$

which is obtained after Taylor expansion of Equation (5) up to first order in the (dimensionless) quantity $k_{r,c}\left(t-\tilde{t}\right)$. The dimensionless and time-independent constant γ involved in Equation (8) satisfies

(9) $$\gamma =1+e^{-k_{r,c}\tilde{t}}\left(\beta -k_{r,c}\int_{0}^{\tilde{t}}{\bar{c}}_{p,c}\left(t\right)e^{k_{r,c}t}dt\right)$$

Another limiting expression of practical interest for $Lum_{c}\left(t\right)$ can be found for observation timescales *t* where processes leading to luciferase inhibition are not significantly operative, i.e., $k_{r,c}t<<1$. In this time domain, the result then reads as

(10) $$Lum_{c}\left(t\right)\approx k_{\nu }V_{T}K_{\mathrm{Hi},c}^{-1}k_{f,c}\tau_{q}c_{p,c}^{\max}\left[{\bar{c}}_{p,c}\left(t\right)+\beta \right]$$

which states that $Lum_{c}\left(t\right)$ at sufficiently short time *t* scales directly with the cell photoactivity profile ${\bar{c}}_{p,c}\left(t\right)$ translated upwards by the β factor defined by Equation (6). All in all, Equations (8) and (10) reveal specific properties of bioluminescence signals by constitutive cells depending on *t*. As discussed in §4, these properties predicted by theory are well supported by experiments, and they further serve to constrain the evaluation of ${\bar{c}}_{p,c}\left(t\right)$ from measured bioluminescence over time (cf. §3.3).

### 3.2. Comparison with Bioluminescence Expression for Metal-Inducible Whole-Cell Lux-Biosensor

The expression formulating the time-dependence of the bioluminescence $Lum\left(t\right)$ (in counts s^−1^) generated at *t* by specific metal (M)-inducible whole-cell biosensors reads as [39,40]

(11) $$Lum\left(t\right)=k_{\nu }V_{T}S_{a}K_{\mathrm{Hi}}^{-1}k_{f}\tau_{q}c_{p}^{\max}J_{u,M}\left[F\left(t\right)⊗{\bar{c}}_{p}\left(t\right)\right]$$

which, for the sake of simplicity, refers here to a monomodal bioluminescence signal. The reader is referred to [40] for extension of Equation (11) to multimodal biosensor response over time.

Equation (11) holds within the Henry M-biouptake regime and the linear Hill gene expression domain that guarantees linearity of $Lum\left(t\right)$ at any *t* versus total bulk metal concentration in solution, hereafter denoted as $c_{M}^{*}$, in accordance with experimental observation [39,40]. Equation (11) is further valid under non-depletive bulk metal conditions, in the absence of significant passive metal biosorption, and considering the initial boundary $Lum\left(t=0\right)=0$, which is in line with the introduction of M in the medium at t=0 [23,39,48]. In Equation (11), $K_{\mathrm{Hi}}$ (in mol m^−3^), $k_{f}$ (mol m^−3^ s^−1^), $c_{p}^{\max}$ (m^−3^) and ${\bar{c}}_{p}\left(t\right)$ (dimensionless) pertaining to metal-sensing cells are the analogues of $K_{\mathrm{Hi},c}$, $k_{f,c}$, $c_{p,c}^{\max}$ and ${\bar{c}}_{p,c}\left(t\right)$ defined in §3.1 for non-specific constitutive biosensors, respectively. $K_{\mathrm{Hi}}$ relates to the dissociation constant between metal-inducible promoter and M-P_reg_ complex, with P_reg_ the regulatory proteins [39,53,54]. $J_{u,M}$ (mol m^−2^ s^−1^) in Equation (11) corresponds to the uptake flux of bioavailable metal ions, which includes contributions of both free metal species and labile metal complexes, as extensively discussed in [40]. It is stressed that the expression of $J_{u,M}$ given in [40] involves the defining features of metal speciation dynamics, beyond the oversimplified equilibrium framework of the Biotic Ligand Model (BLM) and the cognate Free Ion Activity Model (FIAM) [30,32,36,37,38,40]. Within the Henry biouptake regime, the flux $J_{u,M}$ is proportional to $c_{M}^{*}$, and the proportionality coefficient encompasses metal internalisation kinetics and metal speciation/bioavailability parameters [40]. Lastly, the time-dependent function $F\left(t\right)$ in Equation (11) provides a rationale for the nonlinear coupling between the dynamics of M partitioning at the interface between photoactive cell sensor and aqueous medium, and the dynamics of intracellular processes leading to light emission [39]. As such, $F\left(t\right)$ integrates the key timescales that relate to the production of M-P_reg_ complexes (from the speciation-dependent bioaccumulation of M to the very intracellular recognition of M by P_reg_) and to luciferase activity inhibition. The latter is subsumed in a kinetic constant denoted as $k_{r}$ (in s^−1^), which is the equivalent of $k_{r,c}$ for constitutive whole-cell biosensors. 

Comparison between Equations (11) and (5) highlights striking differences in the mathematical structure of the expressions that capture the dependence of metal-responsive and constitutive cell signals on time, especially with regard to the way signals are mediated by the corresponding cell photoactivity profiles ${\bar{c}}_{p}\left(t\right)$ and ${\bar{c}}_{p,c}\left(t\right)$, respectively. These differences originate from the distinct promoter-activation modes characteristic of the metal-sensing (non-constitutive) and constitutive cells: the extent of the ‘off-to-on’ transition of the promoter within an infinitesimal time interval dt is tied to the magnitude of $S_{a}K_{\mathrm{Hi}}^{-1}J_{u,M}c_{p}\left(t\right)dt$ (dimensionless) for the former biosensor type and to that of $K_{\mathrm{Hi},c}^{-1}c_{p,c}\left(t\right)$ (dimensionless) for the latter. Despite these intrinsic differences, the purpose of the coming developments is to analyse the level of similarity between cell photoactivities ${\bar{c}}_{p,c}\left(t\right)$ and ${\bar{c}}_{p}\left(t\right)$ as derived from constitutive and non-constitutive bioluminescent responses measured in media defined by variable nutritional quality.

### 3.3. Inferring Time-Dependent Photoactivity of Constitutive Cells from Their Bioluminescence Signal

The procedure followed to estimate cell photoactivity ${\bar{c}}_{p,c}\left(t\right)$ from the measured time-dependence of bioluminescence generated by constitutive cells, $Lum_{c}\left(t\right)$, is inspired by that detailed in [40] for metal-sensing cells. It relies on proper signal normalization and time-deconvolution. Namely, using Equation (5), the ratio between $Lum_{c}\left(t\right)$ and $Lum_{c}\left(t=t_{\mathrm{ref}}\right)=Lum_{c,\mathrm{ref}}$, where $Lum_{c,\mathrm{ref}}$ is the bioluminescence measured at a selected timepoint, $t=t_{\mathrm{ref}}$, satisfies the expression

(12) $$\frac{Lum_{c}\left(t\right)}{Lum_{c,\mathrm{ref}}}=\frac{{\bar{c}}_{p,c}\left(t\right)+\beta e^{-k_{r,c}t}-k_{r,c}\chi_{c}\left(t\right)⊗{\bar{c}}_{p,c}\left(t\right)}{{\bar{c}}_{p,c}\left(t_{\mathrm{ref}}\right)+\beta e^{-k_{r,c}t_{\mathrm{ref}}}-k_{r,c}\chi_{c}\left(t_{\mathrm{ref}}\right)⊗{\bar{c}}_{p,c}\left(t_{\mathrm{ref}}\right)}$$

which offers the advantage to eliminate the prefactor $k_{\nu }V_{T}K_{\mathrm{Hi},c}^{-1}k_{f,c}\tau_{q}c_{p,c}^{\max}$ in Equation (5). Following [39], we further define ${\bar{c}}_{p,c}\left(t\right)$ as a linear combination of *N* time-dependent sigmoids, $f_{j=1,\ldots ,N}\left(t\right)$, constructed from either the Gompertz model [57] or with help of the error function (erf), i.e.,

(13) $${\bar{c}}_{p,c}\left(t\right)=\sum_{j=1}^{N}r_{j}f_{j}\left(t\right)/\alpha$$

where $r_{j=1,\ldots ,N}$ are scalars independent of time, $f_{j=1,\ldots ,N}\left(t\right)$ satisfy the equalities $f_{j=1,\ldots ,N}\left(t=0\right)=0$, so that ${\bar{c}}_{p,c}\left(t=0\right)=0$, and $f_{j=1,\ldots ,N}\left(t\to \infty \right)=1$. Unless otherwise specified, α in Equation (13) is a normalization constant chosen to ensure the condition ${\bar{c}}_{p,c}\left(t\to t_{\mathrm{last}}\right)\to 1$, with $t_{\mathrm{last}}$ the time at which the last bioluminescence measurement is performed ($t_{\mathrm{last}}=$ 48 h under the conditions of interest here). Theoretical reconstruction of the measured time-dependent ratio $Lum_{c}\left(t\right)/Lum_{c,\mathrm{ref}}$ using Equations (12) and (13) was then realized upon adjustment of $r_{j=1,\ldots ,N}$, β, $k_{r,c}$ and the set of parameters (3 per sigmoid) that describe the shape of the $f_{j=1,\ldots ,N}\left(t\right)$ sigmoids and the time-domain where they are operational. A code written in PTC Mathcad Prime calculus environment was developed for that purpose, with (i) an optimized adjustment of parameters achieved by Levenberg–Marquardt solving algorithm, and (ii) an evaluation of the integral involved in the convolution product using adaptative quadrature method. Each bioluminescence signal subjected to theoretical analysis consisted in ca. 580 data points. Constrained parameter optimization was further added from the separate estimations of $k_{r,c}$ and β on the basis of the simplified forms of $Lum_{c}\left(t\right)$ given by Equations (8) and (10) that were used to fit measured bioluminescence data (after normalization by $Lum_{c,\mathrm{ref}}$) satisfying the conditions $\tilde{t}<t<<\tilde{t}+1/k_{r,c}$ and $k_{r,c}t<<1$, respectively (cf. §3.1). For some of the medium conditions tested, the signal $Lum_{c}\left(t\right)$ decreased abruptly with time, a feature we captured by adding to the sum in Equation (13) a term of the form $g_{N+1}\left(t\right)=-r_{N+1}\left(1-e^{-\left(t-\tau_{N+1}\right)/\alpha_{N+1}}\right)$, where $r_{N+1}$, $\tau_{N+1}$, and $\alpha_{N+1}$ basically quantify the contribution of $g_{N+1}\left(t\right)$ to the signal magnitude, the time position where $Lum_{c}\left(t\right)$ starts to decrease with *t*, and the abruptness of that decrease, respectively. Overall, excellent reconstruction of measured bioluminescence signals could be achieved with N=3 to 5 depending on the medium conditions considered. Clearly, once the time-dependent ratio $Lum_{c}\left(t\right)/Lum_{c,\mathrm{ref}}$ is fitted according to Equations (12) and (13), non-normalized $Lum_{c}\left(t\right)$ can be compared with the one derived from theory after multiplication of $Lum_{c}\left(t\right)/Lum_{c,\mathrm{ref}}$ by $Lum_{c,\mathrm{ref}}$.

## 4. Results and Discussion

### 4.1. Description of the Time-Dependent Response of Non-Specific rrnB P1-luxCDABE E. coli Sensors versus Nutritional Medium Conditions

**Figure 1:**
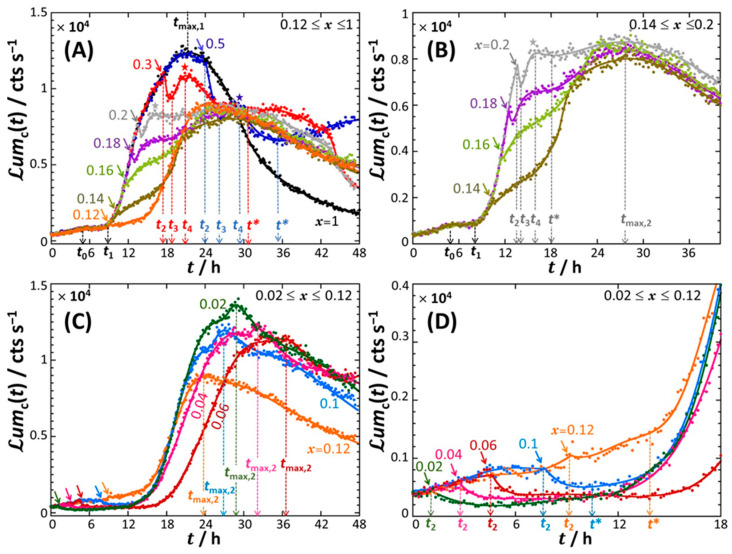
Dependence of the bioluminescence $Lum_{c}\left(t\right)$ on time *t* measured for the constitutive *rrnB* P1-*luxCDABE E. coli* biosensor in nGGM media supplemented with 0.1% tryptone and different concentrations of glucose and xylose, as subsumed in the sugar concentrations ratio *x* (specified) with 0.02 ≤ *x* ≤ 1, at a total Cd concentration $c_{\mathrm{Cd}}^{*}=$ 20 nM. For the sake of clarity, results are shown for 0.12 ≤ *x* ≤ 1 in (**A**) and are zoomed in (**B**) for 0.14 ≤ *x* ≤ 0.2 and *t* ≤ 40 h. Panel (**C**) displays the biosensor response collected for 0.02 ≤ *x* ≤ 0.12 and panel (**D**) provides a zoom of that response for *t* ≤ 18 h. In (**A**–**D**), colored symbols (filled circles) are measurements, and corresponding solid lines are theoretical reconstructions following the procedure detailed in §3.3 (Equations (12) and (13)). The nomenclature adopted here to detail the time-dependent patterns of the biosensor response (i.e., the colored stars and arrows, and the timepoints *t*_0_, *t*_1_, *t*_2_, *t*_3_, *t*_4_, t*, *t*_max,1_ and *t*_max,2_) is specified and further defined in the text. For the sake of data readability, timepoints are only positioned here for selected signals corresponding to *x* = 0.5 (blue) and 0.3 (red) (panel (**A**)), *x* = 0.2 (grey) (panel (**B**)), whereas signal annotations with relevant timepoints *t*_2_ and *t*_max,2_ are provided in (**C**) and (**D**) for all *x*-conditions reported therein (and t* for the only signals *x* = 0.12 and 0.1). Figure 2 reports the dependence of the maxima in bioluminescence on *x* and bulk Cd concentration. Figure 3 specifies the complete assignment of the relevant timepoints *t*_0_, *t*_1_, *t*_2_, *t*_3_, *t*_4_, t*, *t*_max,1_ and *t*_max,2_ for all *x* conditions and Cd concentrations tested.

Figure 1 shows the bioluminescence response $Lum_{c}\left(t\right)$ of constitutive *rrnB* P1-*luxCDABE E. coli* biosensor in nGGM-0.1% tryptone media with varied concentrations of glucose (G) and xylose (X), as indicated by the parameter *x* = [G]/([G] + [X]). The results are provided here for a total Cd concentration $c_{\mathrm{Cd}}^{*}$ given by $c_{\mathrm{Cd}}^{*}=$ 20 nM.

In the absence of xylose (*x* = 1), $Lum_{c}\left(t\right)$ increases slightly with time *t* for $0<t<t_{0}\approx$ 4.5 h and then levels off for $t_{0}<t<t_{1}\approx$ 8.5 h at a plateau value of ca. 0.2 × 10^4^ counts s^−1^ (Figure 1A, black-colored curve). For $t>t_{1}$, $Lum_{c}\left(t\right)$ takes the form of a bell-shaped signal with a ca. 1.25 × 10^4^ counts s^−1^ maximum reached at $t=t_{\max,1}\approx$ 21 h. The peak is asymmetric, and the long-term decay of the bioluminescence is so slow that $Lum_{c}\left(t\right)$ does not reach the value of 0 over the complete duration of the bioassay (Figure 1A). By decreasing the concentration of glucose from *x* = 1 to *x* = 0.5 (blue curve), the signal remains identical to that obtained for *x* = 1 up to ca. $t=t_{2}\approx$ 24 h (marked by a blue arrow in Figure 1A), both with respect to magnitude and shape. For $t_{2}<t<t_{3}\approx$ 26 h, $Lum_{c}\left(t\right)$ decreases abruptly with time, then increases to reach a local maximum at $t=t_{4}\approx$ 29 h (marked by a blue star in Figure 1A) and finally slightly increases for $t>t^{*}\approx$ 35 h. With further decreasing *x* from 0.5 to 0.3 (red curve), the characteristic timescale $t_{2}$ (red arrow in Figure 1A) introduced above to mark the onset of the abrupt decrease in bioluminescence, is now shifted to a lower value ($t_{2}\approx$17.6 h at *x* = 0.3), and the sudden decrease in bioluminescence now spans over a narrow time range, from $t_{2}\approx$ 17.6 h to $t_{3}\approx$ 18.6 h. The signal at *x* = 0.3 subsequently reaches a local maximum at $t_{4}\approx$ 21 h (marked by a red star in Figure 1A) before levelling off at $t=t^{*}\approx$ 30 h and decreasing for t> 43 h. 

At lower glucose contents (or, equivalently, larger xylose concentrations), i.e., for 0.12 ≤ *x* ≤ 0.2 (Figure 1A and zoom in Figure 1B), the characteristic timepoint $t_{2}$ (marked by colored arrows in Figure 1A,B) clearly decreases with decreasing *x*, whereas the signal for $t<t_{2}$ remains strictly similar to that measured at *x* ≥ 0.3. In addition, the length $t_{3}-t_{2}$ of the interval $t_{2}<t<t_{3}$ where $Lum_{c}\left(t\right)$ drops sharply with *t*, is significantly reduced with decreasing *x*. In turn, this specific emission regime becomes difficult to identify at *x* = 0.16, and even more so at *x* = 0.14 and *x* = 0.12 (Figure 1A,B). For these two latter conditions, the characteristic timepoint $t_{3}$ coincides with $t_{2}$, and the timepoint $t_{4}$ also effectively coincides with $t_{2}$ as there is no local maximum to be clearly recognized in the signals. In turn, the well-defined local maxima detected at $t=t_{4}$ for *x* = 0.5 and 0.3 (cf. corresponding colored stars in Figure 1A) leave place at lower *x* to a local shallow maximum (observed for *x* = 0.2, grey star in Figure 1A,B), and to a quasi-plateau (*x* = 0.18) or an increase in bioluminescence with *t* (*x* = 0.16 and 0.14) both spanning over the range $t_{4}<t<t*\approx$ 18 h (Figure 1B). These signal modifications precede the establishment of a maximum in bioluminescence at $t=t_{\max,2}\approx$ 24 h–28 h for 0.12 ≤ *x* ≤ 0.2 (Figure 1A,B). The amplitude of that maximum as counted from the signal value at, e.g., $t=t_{4}$ taken as a reference, i.e., $Lum_{c}\left(t=t_{\max,2}\right)-Lum_{c}\left(t=t_{4}\right)$, increases noticeably with decreasing *x*. Remarkably, the signal decreases linearly with time for $t>t_{\max,2}$ (Figure 1B) and the associated slope is basically independent of *x* for the range 0.12 ≤ *x* ≤ 0.2. 

With a further decrease in *x* from 0.12 to 0.02 (Figure 1C,D), the long-term signal still exhibits a peak for $t\geq t^{*}$ whose maximum is reached at $t_{\max,2}\approx$ 24 h, 27 h, 37 h, 32 h and 29 h for *x* = 0.12, 0.1, 0.06, 0.04 and 0.02, respectively. In addition, with decreasing *x* the bioluminescence is shifted to larger values within the time range $t>t_{\max,2}$ where $Lum_{c}\left(t\right)$ decreases linearly with *t* (linearity evidently applies for *x* = 0.12, 0.10, 0.06 and 0.04). Most interestingly, Figure 1C,D show that the short-term response (t< 10 h) is significantly affected upon decreasing *x* from 0.12 to 0.02, a feature that was not observed for signals measured at larger glucose concentrations. Namely, the timepoints $t_{0}$ and $t_{1}$ defined in Figure 1A,B for 0.12 < *x* ≤ 1 become immaterial for *x* ≤ 0.12 as the corresponding cell signals in the early stage of the response now first increase slightly with time before decreasing sharply beyond a timepoint (marked by the colored arrows in Figure 1C,D) that labels a discontinuity of the signal derivative with respect to *t*. This timepoint actually corresponds to the characteristic time $t_{2}$ (Figure 1D) introduced in Figure 1A,B, with the difference that the abrupt decrease in $Lum_{c}\left(t\right)$ at $t=t_{2}$ now occurs within the first hours (<10 h) of the response. Quantitatively, by decreasing *x* from 0.12 to 0.02, $t_{2}$ decreases from ca. 9 h to 1 h, whereas the envelope of the signal over the range $0<t<t_{2}$ remains the same under all *x*-conditions tested (Figure 1D). Similarly, the time point $t^{*}$ that positions the foot of the maximum reached at $t=t_{\max,2}$ is shifted to lower values with decreasing *x* (Figure 1D).

As an intermediate conclusion, Figure 1 highlights a peculiar typology of the constitutive cell signals with varying the concentration and source of carbon in the medium, namely: a specific short-term response ($t_{0,1,2}$) depending on *x*, the occurrence of an abrupt decrease (hereafter referred to as ‘truncation’) in bioluminescence at $t=t_{2}$ either in the short-term or long-term emission regimes, the presence of a maximum ($t_{\max,1}$, $t_{\max,2}$) and/or a local maximum ($t_{4}$), and a linear decrease in the signal in the long-term for many of the examined situations.

**Figure 2:**
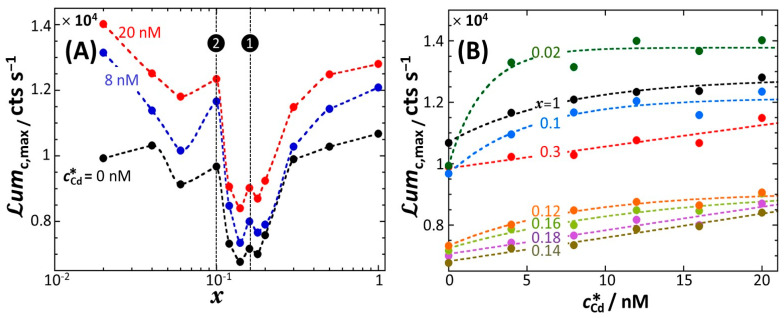
Dependence of the maximum ($Lum_{c,\max}$) of the bioluminescence signals reported in Figure 1A–C on *x* for selected values of $c_{\mathrm{Cd}}^{*}$ (indicated) (**A**), and dependence of $Lum_{c,\max}$ on $c_{\mathrm{Cd}}^{*}$ for selected values of *x* (indicated) (**B**). Symbols are measurements, and dotted lines are guides to the eye. The color-based nomenclature in (**B**) is identical to that adopted in Figure 1. The dotted lines ❶ and ❷ in (**A**) position the characteristic local extrema observed in the dependence of $Lum_{c,\max}$ on *x*. See text for details.

Figure 2 reports the maximum value of bioluminescence measured over the complete duration of the bioassay, denoted hereafter as $Lum_{c,\max}$, as a function of *x* and total Cd concentration $c_{\mathrm{Cd}}^{*}$. At fixed value of *x*, $Lum_{c,\max}$ increases with $c_{\mathrm{Cd}}^{*}$ (Figure 2A), which may be counterintuitive given that *rrnB* P1 promoter is not inducible by Cd. This finding actually relates to Cd-induced hormesis effects. Hormesis refers to the response of many living organisms, including bacteria, to a large spectrum of chemicals found to exert opposite effects at low and high doses. It is generally viewed as an adaptive or overcompensation cell defence mechanism aimed at recovering the cell state prior to exposure to the chemical [58]. The rate of variation of $Lum_{c,\max}$ when changing the glucose-to-xylose concentration ratio remains independent of $c_{\mathrm{Cd}}^{*}$ (Figure 2A). Overall, $Lum_{c,\max}$ decreases with decreasing *x*; it passes through a minimum at x= 0.14 and basically increases with further decreasing *x* to 0.02 (Figure 2A). This minimum coincides with the condition of x marking the transition between time-dependent cell response featuring an abrupt decrease (or truncation) in the signal, either in the long-term emission (i.e., $t_{2}>$ 10 h, Figure 1A,B) or in the short-term emission mode positioned at the foot of the bioluminescence peak located at $t=t_{\max,2}$ (i.e., $t_{2}<$ 10 h, Figure 1C,D). Interestingly, closer inspection of Figure 2A reveals the presence of local extrema in the dependence of $Lum_{c,\max}$ on x, as materialized by the dotted lines ❶ and ❷ in Figure 2A at x= 0.16 and x= 0.1, respectively. The extremum ❶ corresponds to the nutritional condition where the local maximum in $Lum_{c}\left(t\right)$ observed at x= 0.5 and 0.3 (marked by a blue and red star in Figure 1A, respectively) is entirely replaced by the time-domain where $Lum_{c}\left(t\right)$ increases with t for $t_{4}<t<t*$ (Figure 1B). The other extremum ❷ is associated with the cell signal defined by a significant truncation in the short-term emission regime (t< 10 h) (Figure 1D). As a last remark, Figure 2B evidences that $Lum_{c,\max}$ is most significantly impacted by Cd at low $c_{\mathrm{Cd}}^{*}$, as judged by the trends observed for the extremes x= 1 and x= 0.02. This finding is consistent with the hormesis effects invoked above. Additionally, the magnitude of these effects is clearly depending on x. This is in line with the idea that any adaptation of the biosensors to prevent cell damage by Cd is subject to the use of bioavailable and metabolizable nutriments.

**Figure 3:**
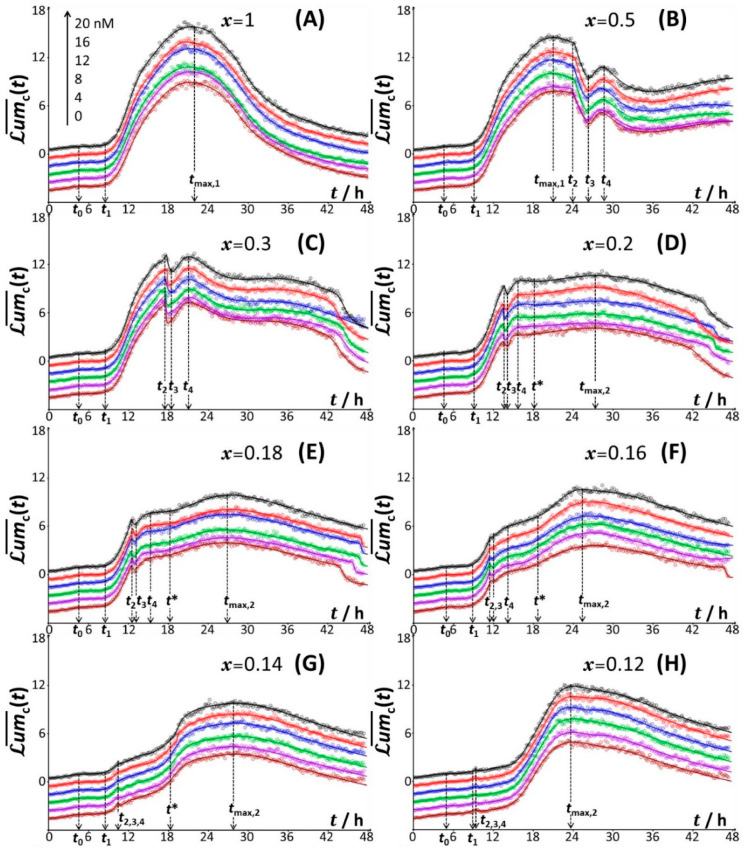

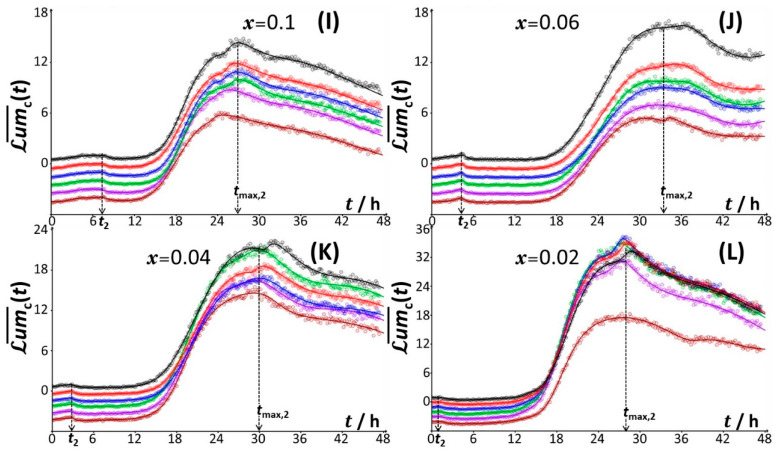
Dependence of the dimensionless bioluminescence ${\bar{Lum}}_{c}\left(t\right)=Lum_{c}\left(t\right)/Lum_{c}\left(t=t_{\mathrm{ref}}\right)$ for the constitutive *rrnB* P1-*luxCDABE E. coli* biosensor on time *t* for different concentrations of glucose and xylose, as subsumed in the parameter *x* with 0.02 ≤ *x* ≤ 1 (indicated). $t_{\mathrm{ref}}$ corresponds here to the timepoint where $Lum_{c}\left(t\right)$ is maximal within the time domain 0 to 10 h. Results are given for different total concentrations of Cd, $c_{\mathrm{Cd}}^{*}$, in the range 0–20 nM and the color nomenclature specified in panel (**A**) for $c_{\mathrm{Cd}}^{*}$ also applies to panels (**B**–**L**). For the sake of clarity and curves readability, ${\bar{Lum}}_{c}\left(t\right)$ profiles corresponding to $c_{\mathrm{Cd}}^{*}=$16, 12, 8, 4 and 0 nM are shifted downwards by −1, −2, −3, −4 and −5, respectively. In (**A**–**L**), symbols (empty circles) are measurements, and plain lines are theoretical reconstructions following the procedure detailed in §3.3 (Equations (12) and (13)). The nomenclature adopted to detail the time-dependent patterns of the constitutive biosensor response (i.e., the timepoints *t*_0_, *t*_1_, *t*_2_, *t*_3_, *t*_4_, t*, *t*_max,1_ and *t*_max,2_) is here systematically specified for each *x*-condition.

For the sake of completeness, we report separately in Figure 3 the time-dependent responses of the constitutive biosensor for each *x*-condition tested and for the various adopted Cd concentrations in the range 0 to 20 nM (Figure 3A–L). For readability purposes, signals are here presented in the dimensionless form defined by ${\bar{Lum}}_{c}\left(t\right)=Lum_{c}\left(t\right)/Lum_{c}\left(t=t_{\mathrm{ref}}\right)$ (cf. §3.3) with $t_{\mathrm{ref}}$ the time point where $Lum_{c}$ is at its maximum in the range 0<t< 10 h. For each condition of *x*, we further systematically specify the positioning of the relevant timepoints *t*_0_, *t*_1_, *t*_2_, *t*_3_, *t*_4_, t*, *t*_max,1_ and/or *t*_max,2_ introduced above to describe the signal patterns featured in Figure 1. The new finding inferred from Figure 3 is that the shape of the cell signal, its defining properties (including, e.g., the presence/absence of truncation in either the short-term or long-term response, or the positioning of the local and/or overall maxima depending on *x*) are not affected by $c_{\mathrm{Cd}}^{*}$. This conclusion conforms to that derived from the consideration of the only maximal signal amplitude in Figure 2A. In turn, the measurements performed at different values of $c_{\mathrm{Cd}}^{*}$ support the repeatability of the time-dependent bioluminescence data, and they ensure that the remarkable evolution of $Lum_{c}\left(t\right)$ with time and *x*, as discussed in Figure 1, is not an artifact. 

Figure 3 further illustrates the successful reconstruction of the biosensor signals collected under all measuring conditions on the basis of the theoretical formalism detailed in §3.1 and §3.3. In the next section, the mechanisms underlying the connections between cell response typology (Figure 1 and Figure 3) and cell metabolism at stake are discussed together with the outcome of the modelling.

### 4.2. Rationale for the Dependence of Constitutive Cell Response on Time

**Figure 4:**
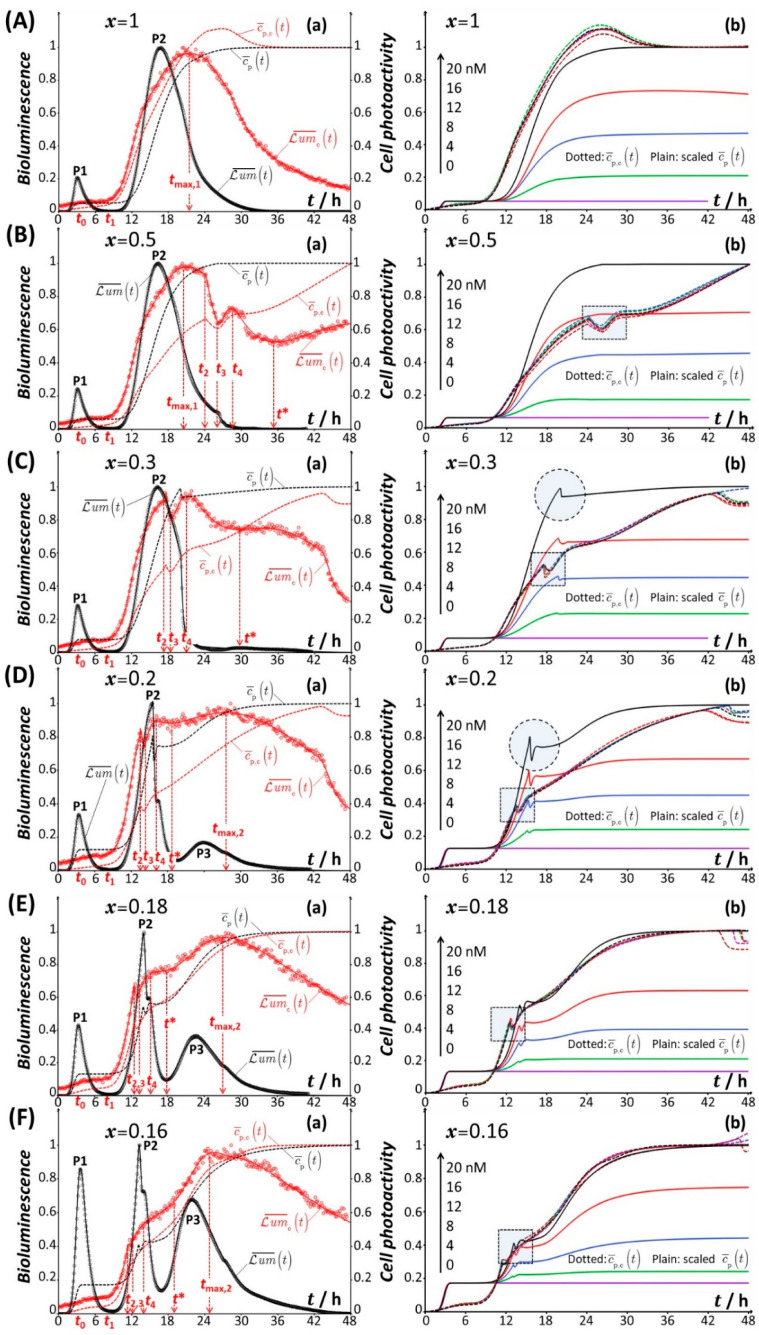

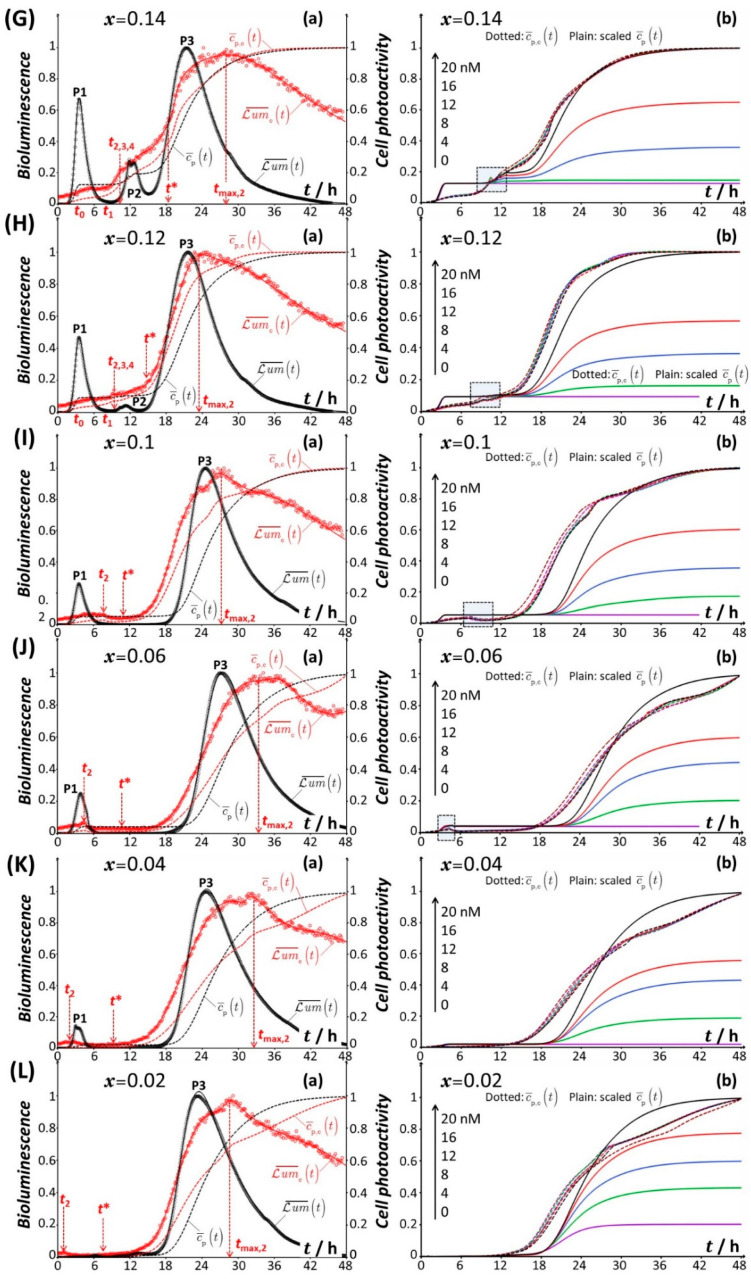
(**a**) Comparison beween dimensionless time-dependent bioluminescence for constitutive *rrnB* P1-*luxCDABE* (${\bar{Lum}}_{c}\left(t\right)$, red color) and non-constitutive Cd-reponsive ($\bar{Lum}\left(t\right)$, black color) *E. coli* whole-cell biosensors at $c_{\mathrm{Cd}}^{*}=$20 nM for different values of *x* (0.02 ≤ *x* ≤ 1, indicated) with corresponding (dimensionless) cell photoactivity ${\bar{c}}_{p,c}\left(t\right)$ and ${\bar{c}}_{p}\left(t\right)$ (red and black dotted lines, respectively). Plain lines are theoretical reconstructions of the bioluminescence profiles following the procedure detailed in §3.3 (Equations (12) and (13)) for the constitutive cells (red) and the procedure given in [40] for the metal-responsive cells (black, Equation (11) in §3.2). Black and red empty circles correspond to measurements. (**b**) Cell photoactivities ${\bar{c}}_{p,c}\left(t\right)$ (dotted lines) and ${\bar{c}}_{p}\left(t\right)$ (plain lines) estimated from theoretical analysis of the bioluminescence response of the constitutive and non-constitutive cells, respectively, for the different total Cd concentrations tested (indicated by the scale arrow) at selected *x* (indicated). For comparison purpose, the ${\bar{c}}_{p}\left(t\right)$ reported in [40] for different $c_{\mathrm{Cd}}^{*}$ in the range 0–20 nM are all multiplied by the factor $1/{\bar{c}}_{p}\left(t=48h\right)$ estimated at $c_{\mathrm{Cd}}^{*}=$ 20 nM, and bioluminescence cell signals are here normalized with respect to their respective maximal values. Panels (**A**–**L**) refer to the different values of *x* of interest in this work (indicated). P1, P2, and P3 correspond to the characteristic bioluminescence peaks detected in the signal of the Cd-responsive biosensor. The nomenclature adopted to detail the time-dependent response patterns of the constitutive biosensor (i.e., the timepoints *t*_0_, *t*_1_, *t*_2_, *t*_3_, *t*_4_, t*, *t*_max,1_ and/or *t*_max,2_) is specified for each *x*-condition. Filled dotted circles and filled dotted squares drawn in some of the panels (**b**) pinpoint remarkable features of ${\bar{c}}_{p}\left(t\right)$ and ${\bar{c}}_{p,c}\left(t\right)$, respectively. See text for details.

With the objective to explain the peculiar variations of $Lum_{c}$ on time *t* and nutritional medium conditions (subsumed in the variable *x*), we provide a thorough comparison between bioluminescence signal for all tested conditions in Figure 4 (Figure 4, panels (a)) and associated cell photoactivity (Figure 4, panels (b)) measured and derived from theory (cf. §3), respectively, for both the constitutive biosensor and its cognate (non-constitutive) Cd-inducible P*zntA*-*luxCDABE Escherichia coli* system. For the latter biosensor type, the experimental and theoretical data refer to those reported and extensively discussed in our previous report [40] for x- and $c_{\mathrm{Cd}}^{*}$-conditions that are identical to those adopted in the current study for the constitutive cells.

To ease comparison, bioluminescence signals in Figure 4 are all normalized with respect to their maximum values, and they are thus reported in the dimensionless form ${\bar{Lum}}_{c}\left(t\right)=Lum_{c}\left(t\right)/Lum_{c,\max}$ and $\bar{Lum}\left(t\right)=Lum\left(t\right)/Lum_{\max}$ for the constitutive and non-constitutive *E. coli* whole-cell biosensors, respectively. As in Figure 3, all relevant timepoints *t*_0_, *t*_1_, *t*_2_, *t*_3_, *t*_4_, t*, *t*_max,1_ and/or *t*_max,2_ are systematically reported in Figure 4. To further compare the corresponding cell photoactivity patterns ${\bar{c}}_{p,c}\left(t\right)$ and ${\bar{c}}_{p}\left(t\right)$ derived from theory (cf. §3) at given x and $c_{\mathrm{Cd}}^{*}$, the values of ${\bar{c}}_{p}\left(t\right)$ reported in Figure 10 of Reference [40] are multiplied here by the quantity $1/{\bar{c}}_{p}\left(t=48h\right)$ evaluated for the situation where $c_{\mathrm{Cd}}^{*}=$ 20 nM. In turn, the so-scaled cell photoactivity ${\bar{c}}_{p}\left(t\right)$ of non-constitutive cells identifies at 20 nM Cd concentration and at t=48 h, to ${\bar{c}}_{p,c}\left(t=48h\right)$, which is unity according to the here-adopted convention (cf. §3.3).

#### 4.2.1. Mechanistic Correspondence between Bioluminescence Signals of Constitutive and Non-Constitutive Cells

We first briefly summarize the main conclusions derived in [40] on the origins of the changes in the response of the non-constitutive (metal-responsive) biosensor with varying *t*, x and $c_{\mathrm{Cd}}^{*}$ (Figure 4, left column, black curves). 

For the situation where the medium supply of glucose to the sensing cells is not limited (x=1), the $\bar{Lum}\left(t\right)$ signal comprises two successive peaks over time, denoted as P1 and P2 (Figure 4A). These peaks define two different light emission regimes: the first regime is driven by the biouptake and utilization of the amino acids bioavailable in solution (P1), and the second follows transitory cell stringent response (i.e., intracellular synthesis of amino acids) (P2). The respective amplitudes of P1 and P2 are further linearly related to $c_{\mathrm{Cd}}^{*}$ for the 0–20 nM range of Cd concentration [40]. By shifting the carbon source gradually from glucose to xylose (Figure 4B,C), the P2 signal exhibits a clear truncation due to a significant decrease in glucose concentration in the medium and to glucose-mediated repression of acetate, a product of the intracellular degradation of glucose. Under such conditions, xylose repression by glucose is also operational. When glucose deficiency in the medium becomes more significant (Figure 4D–H), acetate repression is stopped and acetate then becomes metabolized as described in [40]. This translates into a shouldering (Figure 4D–F) and even a doubling (Figure 4G,H) of peak P2. The modifications of P2 in Figure 4B–H are accompanied by a progressive extinction of the P2 signal component, and full suppression of the latter is achieved at sufficiently low glucose concentrations (Figure 4I–L). The shouldering, doubling, and suppression of P2 with decreasing x from 0.2 to 0.02 are further accompanied by the apparition of a third peak in the long-term response, denoted as P3, whose amplitude increases from x= 0.2 to 0.02 (Figure 4D–L). This third regime of light production is dictated by the metabolization of xylose resource, rendered possible after cells have ended their repression of xylose operative at x> 0.3. Under extreme x-conditions underlying a severe deficiency in glucose (Figure 4J–L), modifications of the short-term P1 response are clearly noticeable with the occurrence of a truncation, doubling, and finally an extinction of peak P1 at x= 0.02. Glucose concentration is then too low to sustain amino acid uptake/utilization and subsequent production of light in this emission regime.

At first glance, Figure 4 evidences that the shape of the bioluminescence response $\bar{Lum_{c}}\left(t\right)$ of constitutive cells, as detailed in Figure 1 and Figure 3 (cf. §4.1), does not share many of the aforementioned markers of the well-identifiable multimodal signals $\bar{Lum}\left(t\right)$ produced by non-constitutive cells (P1-P2, P1-P2-P3, P1-P3 and P3 peak responses operate with decreasing x from 1 to 0.02). Additional differences between $\bar{Lum_{c}}\left(t\right)$ and $\bar{Lum}\left(t\right)$ relate to the initial amplitude of the signal at t= 0 with $\bar{Lum_{c}}\left(t=0\right)\neq 0$ and $\bar{Lum}\left(t\right)=0$. This difference is explained by the distinct functioning principles of both biosensors. Indeed, *rrnB* P1-*luxCDABE* cells are cultured overnight in rich LB growth media (cf. §2.2) prior to bioluminescence measurements. As a result, the intracellular concentration level of luciferase is not 0 immediately after the transfer of the cells into the measuring incubation medium (nGGM-0.1% tryptone of given x) because of the fusion of *rrnB* P1 to the *lux*-operon. Cells have thus already synthesized the required luciferase for light production at t= 0 and, therefore, $\bar{Lum_{c}}\left(t=0\right)\neq 0$, which is in line with observation in [23]. Unlike for constitutive cells, the intracellular concentration of luciferase at t= 0 is 0 for Cd-responsive biosensors because the overnight culture medium is lacking the Cd metal ions that trigger the production of luciferase. The apparent high level of overall dissimilarity between $\bar{Lum_{c}}\left(t\right)$ and $\bar{Lum}\left(t\right)$ signals further echoes the differentiated structure of their defining theoretical time-dependent expressions (cf. Equation (5) versus Equation (11), respectively, and further discussion in §4.2.2).

On the basis of Figure 4 and at the light of the results derived in [40] for the non-constitutive cells, a mechanistic correspondence between the time-dependent patterns $\bar{Lum}\left(t\right)$ and $\bar{Lum_{c}}\left(t\right)$ can be formulated. Namely, the peak P1 (mediated by the uptake/utilization of amino acids from solution) detected for the non-constitutive cells formally corresponds to the early stage response of the constitutive biosensors, i.e., $0\leq t\leq t_{1}$ for 0.12≤x≤1 (Figure 4A–H) and $0\leq t\leq t^{*}$ for 0.04≤x≤0.1 (Figure 4I–K). This first emission regime is poorly resolved for constitutive cells at x=0.02 (Figure 4L), in agreement with the corresponding suppression of peak P1 for their non-constitutive analogues under such x-condition. The peak P2 (that follows stringent control of intracellular proteins synthesis) identifies formally with the response of the constitutive cells over the time range $t>t_{1}$ at x= 1 (Figure 4A) and $t_{1}\leq t\leq t^{*}$ for 0.12≤x≤0.5 (Figure 4B–H). The maximum of that signal component is reached at $t=t_{\max,1}$ (x= 1 and 0.5, Figure 4A,B), and $t=t_{4}$ or $t=t^{*}$ for 0.12≤x≤0.3 (Figure 4C–H). For x≤0.1 (Figure 4I–L), it is virtually impossible to recognize a specific intermediate regime that separates the short- and long-term responses of the constitutive sensors, in agreement with the corresponding suppression of peak P2 for non-constitutive cells. Finally, the analogue of peak P3 associated with xylose metabolism by non-constitutive cells can be clearly tracked in the signal of the constitutive sensors at $t\geq t^{*}$ and x≤0.2 (Figure 4D–L): the maximal amplitude of the signal in this third long-term emission regime is clearly reached at $t=t_{\max,2}$. 

There are additional supports for the sound basis of the above equivalence drawn between $\bar{Lum}\left(t\right)$ and $\bar{Lum_{c}}\left(t\right)$ and for the underlying connections between signal shapes (regardless of their respective complexity) and operational metabolic pathways. Indeed, the truncations of the bioluminescence signals in the first and second emission regimes of metal-responsive cells can be spotted at $t=t_{2}$ (depending on x) for the non-constitutive sensors, with an abrupt decrease in the bioluminescence signal for $t_{2}\leq t\leq t_{3}$. In addition, when glucose deficiency in the medium is significant, the exploitation of acetate by non-constitutive sensors translates into a shouldering/doubling of peak P2, whereas, for constitutive cells, it is actually reflected by an increase in the signal with time for $t_{3}\leq t\leq t_{4}$ (Figure 4D–H). Lastly, the non-monotonous dependence of $Lum_{c,\max}$ on x evidenced in Figure 2 reflects the gradual suppression of the second emission regime (post-stringence) upon decreasing x from 1 to 0.12–0.14 and the growing contribution of the long-term signal (marking cell utilization of xylose) for x running from 0.12–0.14 to 0.02. As argued in [40] for non-constitutive cells, the local extrema highlighted in Figure 2 at intermediate x likely originate from the simultaneous (and not necessarily sequential [59,60]) utilizations of different carbon sources (glucose, xylose and/or acetate) depending on cell energetic trade-offs and demands. These extrema are the analogues of those observed in [40] for the dependence of the peak P1 amplitude on x (see Figure 5A in [40]). 

Overall, Figure 2 therefore combines qualitatively the defining amplitudes of P1, P2, and P3 detailed in [40] for metal-responsive cells upon modulation of the nutritional medium conditions. This parallel between specific and non-specific biosensors that generate very different time-dependent bioluminescence signals is all the more remarkable that their respective sensitivities differ significantly, with a $Lum_{\max}$ that can be up to two orders of magnitude larger than $Lum_{c,\max}$ over the ensemble of medium compositions considered [40]. Such a difference in sensitivity is the result of the distinct engineering of the constitutive and non-constitutive sensing cells, which leads to contrasting amounts of *lux*-reporter genes. As a last remark, we observe from Figure 4 that the timescale and onset of the second and third emission regimes under given x condition is different for both types of sensing cells. This latter aspect is clarified in the next section with the analysis of the time-dependent cell photoactivity profiles derived by time-deconvolution of the measured bioluminescence signals according to Equation (12) (case of constitutive cells) and Equation (11) (case of non-constitutive cells, cf. [40]).

#### 4.2.2. Comparison between Time-Dependent Cell Photoactivity Profiles for Constitutive and Non-Constitutive Cells

The (scaled, cf. introduction of §4.2) cell photoactivity profiles ${\bar{c}}_{p}\left(t\right)$ reported in Figure 4 for metal-responsive sensors at different Cd concentrations in the 0–20 nM range (right column, solid-colored lines) were derived in [40] from deconvolution of the bioluminescence signal following Equation (11). Briefly, for given values of $c_{\mathrm{Cd}}^{*}$ and x, ${\bar{c}}_{p}\left(t\right)$ takes the form of 1, 2, or 3 successive sigmoids depending on the number of detected peaks (P1, P2 and P3). This conversion of bioluminescence peaks into increasing sigmoidal function of time is the direct consequence of the convolution product that underlies the connection between bioluminescence and amount of (photoactive) cells over time. By construction, the ${\bar{c}}_{p}\left(t\right)$ profiles were obtained from the time-deconvolution of the signals normalized by the amplitude of peak P1 (the latter increases linearly with $c_{\mathrm{Cd}}^{*}$) [40]. In turn, this explains why ${\bar{c}}_{p}\left(t\right)$ at fixed value of x does not depend on $c_{\mathrm{Cd}}^{*}$ within the time domain where the first emission regime prevails (see [40] for further details). In the time domains corresponding to the second- and third-emission regimes, ${\bar{c}}_{p}\left(t\right)$ increases at given *t* with $c_{\mathrm{Cd}}^{*}$, which is a consequence of the linear increase in the P2 and P3 amplitudes with increasing $c_{\mathrm{Cd}}^{*}$. Most importantly, in bimodal P1–P2 signal configuration, the truncation of peak P2 due to acetate repression by glucose is materialized by an abrupt decrease in ${\bar{c}}_{p}\left(t\right)$ with time followed by a plateau value reached when bioluminescence approaches 0 value (Figure 4C(b), cf. dotted circle therein). When there is repression of acetate by glucose and a subsequent use of acetate by the cells after that repression is turned off (Figure 4D), the corresponding truncation and shouldering of P2, respectively, lead to a local and abrupt peaked variation of ${\bar{c}}_{p}\left(t\right)$ materialized by the dotted circle in Figure 4D(b). Similar features can be identified when zooming in on the part of the ${\bar{c}}_{p}\left(t\right)$ profiles that correspond to a truncated P1 signal (Figure 4J–K) as a result of early catabolite repression at sufficiently low values of x [40].

Unlike for metal-detecting cells, the cell photoactivity profiles ${\bar{c}}_{p,c}\left(t\right)$ derived for the constitutive sensor using Equations (12) and (13) are basically independent of $c_{\mathrm{Cd}}^{*}$ under all x-conditions tested. This property agrees with the construction of this sensor type and with the normalisation adopted for ${\bar{c}}_{p,c}\left(t\right)$ which leads to scaling of data with respect to metal-induced hormesis effects. All ${\bar{c}}_{p,c}\left(t\right)$ curves feature an increase in the number of photoactive cells over the full duration of the bioluminescence assays, and some are marked by a plateau value reached at large time (Figure 4A,E–I). Exceptions include the conditions x= 0.5, 0.3, 0.2, 0.06, 0.04, and 0.02 (Figure 4B–D,J–L) for which ${\bar{c}}_{p,c}\left(t\right)$ curves do not exhibit such a plateau at the end of the assay. This latter finding is consistent with the absence of a linear decrease in $Lum_{c}\left(t\right)$ with time in the long-term cell response (cf. Equation (8) and validity conditions thereof). In contrast, this linear decrease in $Lum_{c}\left(t\right)$ with time, as predicted by the theory under the conditions underlying the applicability of Equation (8), is very well observed in Figure 4E–I. In addition, conformably to Equation (10) valid at the sufficiently short time *t*, in the initial stage of the response the bioluminescence signals, $Lum_{c}\left(t\right)$ closely follows the ${\bar{c}}_{p,c}\left(t\right)$ profiles within a constant β that defines the amplitude of $Lum_{c}\left(t=0\right)$ (cf. Equation (7)). Most strikingly, specific changes in ${\bar{c}}_{p,c}\left(t\right)$ in the form of local minima or local peaked changes of the profiles (Figure 4B–H, panels (b) and cf. dotted squares therein) are observed within the time domains marking acetate and/or xylose repressions by glucose. These peculiarities of the cell photoactivity patterns ${\bar{c}}_{p,c}\left(t\right)$ are strongly reminiscent of those discussed for their metal-responsive analogues, despite of the pronounced differences in the respective shapes of $Lum_{c}\left(t\right)$ and $Lum\left(t\right)$ signals. This is especially obvious when comparing ${\bar{c}}_{p,c}\left(t\right)$ and ${\bar{c}}_{p}\left(t\right)$ for x=0.18, 0.16, and 0.14 (Figure 4E–G). Under the x-conditions marking a transition between significant suppression and increases in the second and third emission regime, respectively (cf. §4.1), the observation of the aforementioned associated characteristics of ${\bar{c}}_{p,c}\left(t\right)$ becomes more difficult even though data zooming reveal uncontestably their presence. A similar conclusion applies at sufficiently low values of x where the short-term emission regime is gradually supressed with accompanying truncation of both $Lum_{c}\left(t\right)$ and ${\bar{c}}_{p,c}\left(t\right)$ (Figure 4H–J, panels (b), see dotted squares therein). 

The above analogy between ${\bar{c}}_{p,c}\left(t\right)$ and ${\bar{c}}_{p}\left(t\right)$ is a strong marker of the sound foundations of Equation (5) and Equation (11) adopted to interpret the bioluminescence response of engineered bacteria displaying very distinct sensing functionality. Their relative comparison highlights that, e.g., the second emission regime (corresponding to peak P2 for non-constitutive cells) is reached at a shorter time by constitutive cells (Figure 4A) and that acetate ‘’on-off’’ repression processes are achieved by these cells type at lower x and shorter *t* (Figure 4B–H) compared with metal responsive cells. This difference in the delay of apparition of catabolite repression is a result of the different rate of initial luciferase production at t=0 for both sensors (§4.1).

**Figure 5:**
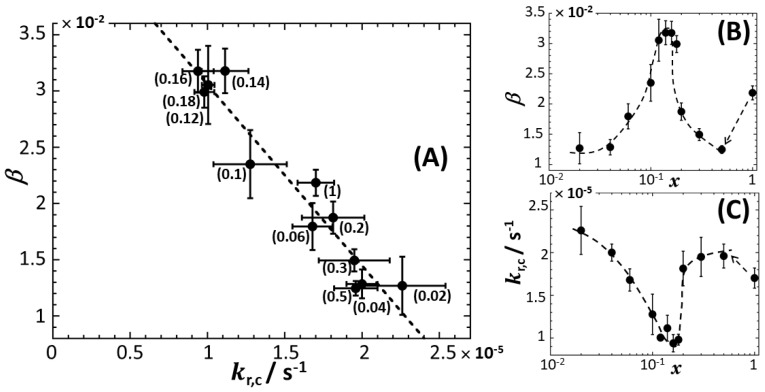
Parameters β (dimensionless) and $k_{r,c}$ (in s^−1^) derived from the analysis of bioluminescence response of constitutive *rrnB* P1-*luxCDABE* whole-cell biosensors using Equations (12) and (13) (cf. §3.3). (**A**) Variation of β versus $k_{r,c}$. Numbers between brackets specify the corresponding value of x. (**B**,**C**) Variations of β and $k_{r,c}$ versus x, respectively. Error bars in (**A**–**C**) refer to dispersions in β and $k_{r,c}$ values estimated from theoretical recovery of the bioluminescence signals measured at different values of the total Cd concentration in the range 0 to 20 nM.

Lastly, in Figure 5, we provide the values obtained for the constant β and $k_{r,c}$ involved in the expression of $Lum_{c}\left(t\right)$ for all medium compositions (Equations (5) and (12)). It is found that $k_{r,c}$ lies in the range ~1 × 10^−5^ s^−1^ to 2.5 × 10^−5^ s^−1^ depending on x, from which we infer that the characteristic timescale for effective light inhibition varies between 11 h and 27 h. This magnitude of $1/k_{r,c}$ is significantly larger than the ~10 to 25 min value of $1/k_{r}$ derived for metal-responsive cells from bioluminescence signal reconstruction on the basis of Equation (11) [40]. Following the reasoning in [40] and referring to the conclusion by Iqbal et al. [61], we argue that such a difference between $1/k_{r,c}$ and $1/k_{r}$ is tied to the extent of luciferase production inhibition by flavine mononucleotide (FMN), a product of the bioluminescence reaction. The more significant is the production of light, the higher is the intracellular FMN concentration, and the more pronounced the inhibition of the bioluminescence reaction becomes. In turn, this converts into a larger (lower) effective kinetic constant (timescale, respectively) for the inhibition of the bioluminescence reaction. This explanation is consistent with the fact that metal-responsive cells generate significantly more light (up to two orders of magnitude more [40]) than their constitutive analogues for a given nutritional medium composition, a feature that is inherent to their genetic construction. In agreement with the behaviour of $Lum_{c}\left(t\right)$ at t=0, β (dimensionless) is not 0 (Equation (7)) and is found to vary between ~1.2×10^−2^ and ~3.2 × 10^−2^ over the range of the x-conditions tested. Figure 5A further evidences an overall decrease in β with $k_{r,c}$.

As argued above, $k_{r,c}$ increases with increasing light production due to enhanced inhibition of bioluminescence reaction by FMN product. Such an increase in $Lum_{c}\left(t\right)$ is necessarily connected to an increase in the kinetic constant $k_{f,c}$ for luciferase production and/or to an increase in the medium carrying capacity $c_{p,c}^{\max}$ (cf. the prefactor in Equation (5)). In turn, using Equation (6) that underpins the dependence $\beta \propto 1/\left(k_{f,c}c_{p,c}^{\max}\right)$, we infer that β should decrease with increasing $k_{f,c}c_{p,c}^{\max}$ or increasing $k_{r,c}$, which is in line with the results displayed in Figure 5A. This inverse relationship between β and $k_{r,c}$ is further illustrated in Figure 5B,C where their respective variations with changing x are plotted separately. These figures show the existence of a minimum (maximum) for $k_{r,c}$ (β, respectively) with decreasing x from 1 to 0.02. This minimum echoes the one observed for $Lum_{c,\max}$ in Figure 2. Again, it is consistent with the idea that bioluminescence reaction inhibition is all the more reduced (with associated decrease in $k_{r,c}$) as light production by cells (and therewith $Lum_{c,\max}$) decreases.

## 5. Conclusions

In this work, we report a detailed analysis pointing the differences and similarities between time-resolved bioluminescence signals produced by specific (non-constitutive) Cd-sensing and non-specific (constitutive) *lux*-based whole-cell bacterial sensors in media differing in terms of source and concentration of carbon. Observed differences apply to the overall shape, number, and time occurrence of bioluminescence peaks, to the positioning of signal discontinuities over time, and to the magnitude of the cell response. The peculiar patterns of the constitutive cell signals are here reconstructed on the basis of an original theoretical model that accounts for the finite delay of bioluminescence emission by luciferase, the characteristic timescale for the inhibition of the bioluminescence reaction, the strength of the constitutive promotor (*rrnB* P1 in this study) and the dependence of cell photoactivity on time. This formalism is the counterpart of the one we reported earlier [39] for metal-responsive whole-cell biosensors. In turn, we evidence here how photoactivity of constitutive cells can be retrieved from proper time-deconvolution of normalized bioluminescence signals for a given nutritional quality of the incubation medium. 

Despite the different modes of promoter activation for constitutive and non-constitutive cells and the apparent non-similarity of their bioluminescence time-response, the dependence of their cell photoactivities on time as retrieved from theory shares remarkable features. In particular, the bioluminescence signals produced by specific and non-specific biosensors under stringence-controlled conditions both correspond to two successive sigmoidal increases in their photoactivity, reminiscent of well-known biphasic cell growth. We further provide mechanistic connections between local extrema and long-term emission observed in the response of constitutive cells, with on-or-off occurrence of acetate and/or xylose repressions by glucose upon varying glucose-to-xylose concentrations ratio. Similar to metal-responsive cells, these catabolite repressions lead to well-identified discontinuities for the time-dependent rate of variation of photoactivity of constitutive cells over the duration of the bioluminescence assays. Differences between the two biosensing systems refer to the delay of the apparition of these discontinuities and to the non-zero bioluminescence measured at a short time for constitutive cells. This is the result of their finite photoactivity (inherent to the nature of their genetic construction) prior to monitoring their response. 

The analysis based on the confrontation between time-resolved bioluminescence measurements and the theory shows that photoactivities of constitutive and metal-responsive *lux*-biosensors depend in a rather similar way on nutritional conditions or, equivalently, on the cell metabolic activity required to sustain light production after promoter activation. This equivalence evidenced between biosensors featuring different promoter actuation mechanisms is basically impossible to sort out from the only inspection of their well-differentiated bioluminescence responses over time. In that respect, the recourse to sound theoretical formalisms for rationalizing signal dependencies on time constitutes an added value to common biosensing practice in environmental and ecotoxicological studies where cell response is often exploited from mere qualitative considerations of the only maximal cell signal amplitude. Future extensions of the here-reported theoretical background may include detailed integrations of transcription-translation processes to model gene regulatory functions and resulting time-dependent concentrations of, e.g., RNA and products from *lux* operon expression. This would surely contribute to increase the biological significance of the here-reported modelling based on the practical use of semi-empirical Hill functions and basic chemical kinetic principles. However, such theoretical efforts should necessarily maintain parsimony in the number of introduced variables to be retrieved from data analysis so as to avoid overparameterization-related issues. The formalism elaborated in this work succeeds in meeting such a compromise while capturing the main properties of the full biosensor’s response and changes thereof with modulation of medium nutritional quality. 

## Data Availability

All raw bioluminescence data reported in this work are available upon request, as is the PTC Mathcad Prime code developed for the theoretical analysis of the time-dependent response of constitutive bioluminescent *E. coli* sensors.
